# Eutectic In Situ Modification of Polyamide 12 Processed through Laser-Based Powder Bed Fusion

**DOI:** 10.3390/ma16052050

**Published:** 2023-03-01

**Authors:** Samuel Schlicht, Dietmar Drummer

**Affiliations:** 1 Institute of Polymer Technology, Friedrich-Alexander-Universität Erlangen-Nürnberg, Am Weichselgarten 10, 91058 Erlangen, Germany; 2 Collaborative Research Center 814, Friedrich-Alexander-Universität Erlangen-Nürnberg, Am Weichselgarten 10, 91058 Erlangen, Germany

**Keywords:** semi-aromatic polyamides, aramides, powder bed fusion, selective laser sintering, in situ modification, eutectic, eutectic polymers

## Abstract

Laser-based powder bed fusion (LPBF) of polymers allows for the additive manufacturing of dense components with high mechanical properties. Due to inherent limitations of present material systems suitable for LPBF of polymers and required high processing temperatures, the present paper investigates the in situ modification of material systems using powder blending of p-aminobenzoic acid and aliphatic polyamide 12, followed by subsequent laser-based additive manufacturing. Prepared powder blends exhibit a considerable reduction of required processing temperatures dependent on the fraction of p-aminobenzoic acid, allowing for the processing of polyamide 12 at a build chamber temperature of 141.5 °C. An elevated fraction of 20 wt% of p-aminobenzoic acid allows for obtaining a considerably increased elongation at break of 24.65% ± 2.87 while exhibiting a reduced ultimate tensile strength. Thermal investigations demonstrate the influence of the thermal material history on thermal properties, associated with the suppression of low-melting crystalline fractions, yielding amorphous material properties of the previously semi-crystalline polymer. Based on complementary infrared spectroscopic analysis, the increased presence of secondary amides can be observed, indicating the influence of both covalently bound aromatic groups and hydrogen-bound supramolecular structures on emerging material properties. The presented approach represents a novel methodology for the energy-efficient in situ preparation of eutectic polyamides, potentially allowing for the manufacturing of tailored material systems with adapted thermal, chemical, and mechanical properties.

## 1. Introduction

Laser-based powder bed fusion (LPBF) of polymers represents a well-established additive manufacturing process, allowing for the additive processing of semi-crystalline and amorphous polymers. Considering the predominant application of isothermal processing strategies, the range of materials suitable for LPBF is limited based on the structural requirement of material-specific thermal processing windows. Therefore, the processing of polymers exhibiting tailored mechanical and thermal properties is inherently restricted by technological and economic considerations. To overcome existing limitations in the range of materials suitable for LPBF, the in situ formation of modified material systems has been described for both solid-based and solid–liquid systems, enabling the adaptation of mechanical and thermal properties of manufactured parts. However, the in situ modification in LPBF remains subject to material-specific, complex processing technology, limiting the present in situ enhancement of material properties. Concerning the overall field of polymer engineering, considerable enhancements of thermal and mechanical properties have been obtained by the introduction of liquid crystalline semi-aromatic material systems, being predominantly used in injection molding of thin-walled components due to reduced melt viscosities and increased operating temperatures. Besides well-described synthesis routes for the preparation of aromatically modified polyamides, produced by melt condensation of monomers [1,2,3,4] and by solution-based [5,6], complex-mediated condensation of various modified aromatic monomers, additive manufacturing applications of aromatically modified polyamides are scarce. Based on technological restrictions and a lacking economic viability of the additive processing of semi-aromatic polyamides, the direct processing of aforementioned semi-aromatic material systems is inherently limited. To overcome limitations in the processing of tailored material systems and to allow for the considerable extension of material systems suitable for LPBF, the transfer of traditional synthesis routes, described in the early development of semi-aromatic and fully aromatic polyamides, as well as novel sustainable synthesis routes on the in situ modification of polyamides, represents a promising approach.

## 2. State of the Art

### 2.1. Thermal Material Properties in Quasi-Isothermal LPBF of Polymers

Quasi-isothermal laser-based powder bed fusion of polymers is characterized by the layer-wise manufacturing of semi-crystalline polymers within a material-specific thermal processing window [7]. To allow for minimized geometric deflections of manufactured geometries, the ambient processing temperature is specified to minimize isothermal material crystallization, allowing for the definition of an isothermal processing window [8], defined based on the crystallization and melting onset of the underlying material. However, the occurrence of isothermal crystallization has been confirmed based on experimental [8] and analytical investigations [9,10]. Considering the significance of the isothermal processing window in the LPBF of polymers, the range of materials applicable for LPBF is inherently restricted. A major limitation is represented by the processing of high-melting, technical polyamides that are subject to material aging [11] such as oxidative degradation [12], leading to altered mechanical properties [13], restricting the recyclability and ecologic viability of polyamides processed at high isothermal temperatures. Furthermore, isothermal crystallization kinetics exhibit a substantial influence on the processability of filled material systems [14,15], implicitly influencing the applicability of modified material systems.

### 2.2. Properties of Eutectic Polymer-Based Compounds

The targeted modification of thermal properties of polymers by means of additives of low molecular mass has been extensively described with regard to deep eutectic solvents (DES) [16,17,18], providing promising, tunable thermal and chemical properties for a variety of applications. Deep eutectic solvents that show structural similarities to ionic liquids rely on the combination of a hydrogen bond acceptor and a hydrogen bond donor, with the hydrogen donor considerably influencing the extent of the melting point reduction [19,20]. Established deep eutectic solvents have been predominantly described based on choline chloride and other quaternary ammonium compounds, with the obtained eutectic systems exhibiting melting point reductions exceeding 100 K relative to the melting point of the reactants [21].

As a sub-class of DES, supramolecular deep eutectic solvents (SUPRADES) [17] based on cyclodextrins [22,23] have been proposed, applying cyclic polysaccharides as hydrogen bond acceptors. By heating prepared dry powder blends, viscous compounds exhibiting good adhesive properties can be obtained from the combination of cyclodextrins and dicarboxylic as well as tricarboxylic acids. Relying on the formation of hydrogen-bonded supramolecular systems [17,22], obtained thermal properties are governed by the formation of hydrogen-bonded supramolecular structures, allowing for the extensive adaption of thermal material properties by adapting the composition of binary material systems. Applying aromatic monomers, de Lacalle et al. [24] described the intermediate formation of deep eutectic monomers, followed by photo-induced polymerization, allowing for tailored material properties with a considerable material toughness.

Based on binary polymer–monomer mixtures, eutectic properties of poly (ε-caprolactone) and trioxane have been described as early as 1977 by Wittmann et al. [25], obtaining a melting peak temperature of 40 °C, demonstrating the targeted modification of thermal properties of polymers using corresponding monomers.

### 2.3. Synthesis Routes of Semi-Aromatic Polymers

Synthesis routes, described for the preparation of semi- and fully-aromatic polyamides, can be predominantly distinguished between the condensation of diamines and dicarboxylic acids and the auto condensation of aromatic aminocarboxylic acids. Commercially available semi-aromatic polymers are prepared based on the condensation of diamines and dicarboxylic acids, being used in the form of carboxylic chlorides and anhydrides. Formed polymers exhibit considerably altered mechanical and thermal properties compared with aliphatic polyamides, being widely applied in high-temperature environments and challenging tribological and mechanical applications. In contrast, synthesis routes based on auto condensation of aminocarboxylic acids have been described based on melt-condensation and solution-based synthesis. Existing synthesis routes applying solution-based auto condensation of aromatic aminocarboxylic acids are predominantly based on applying phenyl phosphines as condensation agents in the presence of tertiary amines and metal salts, initially described by Yamazaki et al. [5]. Obtained polymers exhibit high molecular weights and melting points, depending on applied synthesis conditions, condensing agents, and solvents. Similar synthesis routes, applied by Shibasaki et al. [6] demonstrate the applicability of modified aromatic monomers. By applying the auto condensation of N-alkylated p-aminobenzoic acid with a varying aliphatic chain length, varying thermal properties could be obtained, hence significantly expanding the range of thermal properties accessible through polymers exhibiting a fully aromatic main chain. Given the complexity of solution-based synthesis of fully and semi-aromatic polyamides, various approaches for obtaining semi-aromatic polymers from melt condensation have been proposed. Based on the bulk polymerization of monomers, the formation of non-isomorphic copolymers yields low-melting products while maintaining similar thermal decomposition properties [1]. Similar approaches have been proposed based on solid-state polycondensation [26,27,28,29], obtaining a wide range of accessible thermal properties and molecular weights. However, to date, aforementioned synthesis routes are inherently limited to bulk processing, posing the inherent requirement of further processing steps at elevated processing temperatures.

### 2.4. Thermal Properties of Semi-Aromatic Polyamides

The length of aliphatic monomers and the occurrence of block copolymers in the chain significantly influence the thermal properties of semi-aromatic polyamides. With regard to semi-aromatic polyamides obtained from diamines and dicarboxylic acids, leading to alternating copolymers, considerably elevated melting points and glass transition temperature values can be observed. A decreasing length of the aliphatic monomer, correlated with an increasing mass fraction of aromatic monomers, is correlated with increasing melting points [30]. In contrast, block copolymers, predominantly obtained from melt condensation and solid state condensation [1,2,4,27,28,30,31], exhibit a non-linear thermal dependency of the fraction of aromatic monomers [2,28,32]. An increasing mass fraction of aromatic monomers added to aliphatic polymers results in the formation of block copolymers, implicitly affecting inter-polymer hydrogen bonding [33]. Therefore, comparatively low contents of aromatic monomers contained in block copolymers induce a reduction of the melting point [1]. Endo et al. [34] demonstrated the influence of varying monomers as well as the formation of block copolymers on resulting thermal properties, achieving a significant reduction in observed melting points, hence facilitating the melt processability of the resulting polymers.

## 3. Materials and Methods

### 3.1. Material Preparation and Processing

**Figure 1:**
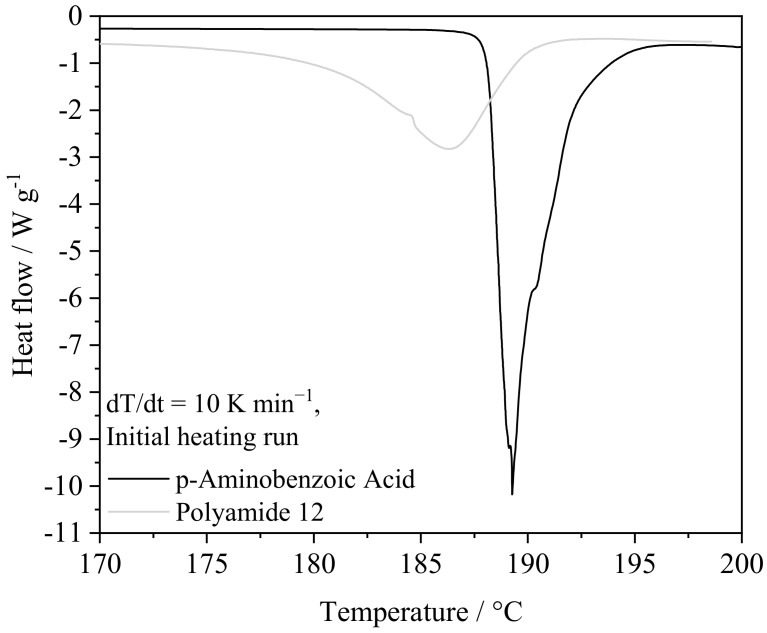
Depiction of differential scanning calorimetric characteristics of PA12 and pABA.

The in situ modification of Polyamide 12 is based on binary powder blends obtained from p-aminobenzoic acid (pABA), Carl Roth GmbH + Co. KG, Karlsruhe, Germany, and Polyamide 12 powder of type PA 2200 (EOS GmbH, Krailling, Germany). To improve the flowability of prepared powder blends and to facilitate the dispersion of pABA particles, 0.5% of fumed silica (Aerosil R 8200, Evonik AG, Duisburg, Germany), corresponding to the mass of pABA, are added. All educts are processed as received. Blending is conducted using a laboratory mixer, applying a batch size of 250 g and a blending time of 15 min at a controlled ambient temperature of 25 °C. A rotational speed of 5000 rpm is kept constant during the blending process. Applied educts exhibit melting peaks of 186 °C (PA12) and 189 °C (pABA) and crystallization peaks (dT/dt = −10 K min^−1^) of 152 °C (PA12) and 155°C (pABA), respectively, indicating a thermal compatibility concerning the isothermal processing window, displayed in Figure 1. The thermal processing window, defined by the model of isothermal laser sintering [8], indicates the presence of a two-phase state of fused, molten, and unexposed, solid material, requiring a considerable thermal difference between the melting temperature and the crystallization onset. The crystallization of pABA exhibits an influence of the previous thermal history, depicting a dependency of the previous heating run regarding both the crystallization enthalpy and the crystallization temperature.

**Figure 2:**
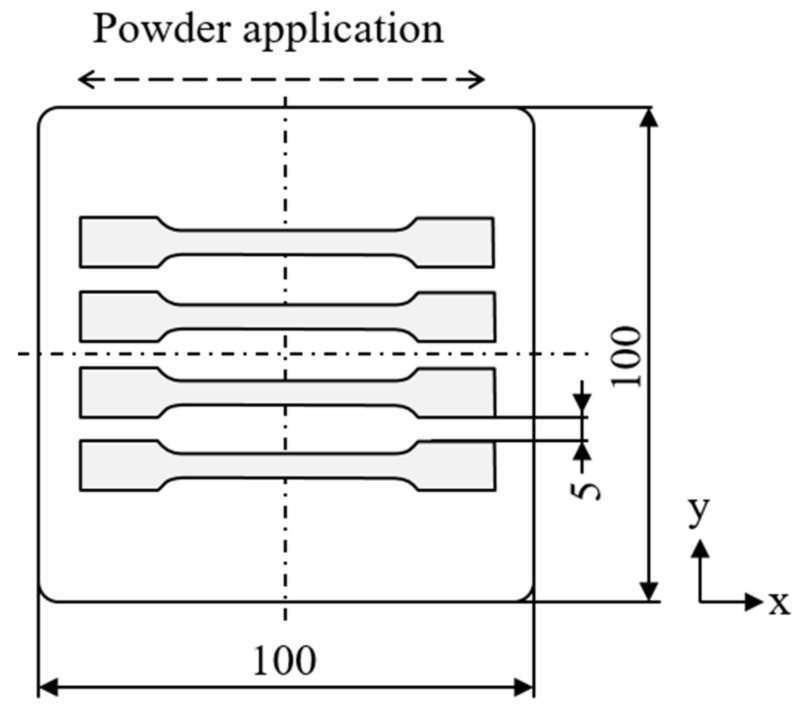
Schematic depiction of the build process layout relative to the powder coating orientation, units in mm.

Manufactured specimens comprise tensile bars (DIN EN ISO 3167:2014-11, type 1A) scaled by a factor of 0.5. All processing is conducted using an LPBF machine of the type EOS P 396 (EOS GmbH, Krailling, Germany) equipped with a 70 W CO_2_ laser with a wavelength of λ = 10.6 μm. A build chamber insert exhibiting an area of 100 × 100 mm^2^ is applied for limiting the required amount of powder blend. Underlying exposure parameters are chosen according to near-optimal parameters for the processing of unmodified PA12, corresponding to a laser power of P = 16.8 W, a hatch distance of d_hatch_ = 0.2 mm, an exposure speed of v = 2000 mm s^−1^, and a layer height of h_Layer_ = 0.12 mm. Alternating meander scanning is specified as the underlying exposure strategy. The underlying process layout is displayed in Figure 2.

The isothermal processing temperature is chosen according to preceding differential scanning calorimetric measurements depending on the isothermal processing window of a specific prepared material system. Following the processing, the powder bed surface is tempered at isothermal conditions for 60 min to allow for the isothermal crystallization of manufactured parts. All materials were processed applying a platform temperature of 100 °C. Employed isothermal powder bed temperatures are chosen based on the preceding differential calorimetric characterization of prepared powder blends, displayed in Table 1. Shown molar fractions correspond to the relation of aliphatic and aromatic monomers.

### 3.2. Powder Blend Characterization

**Figure 3:**
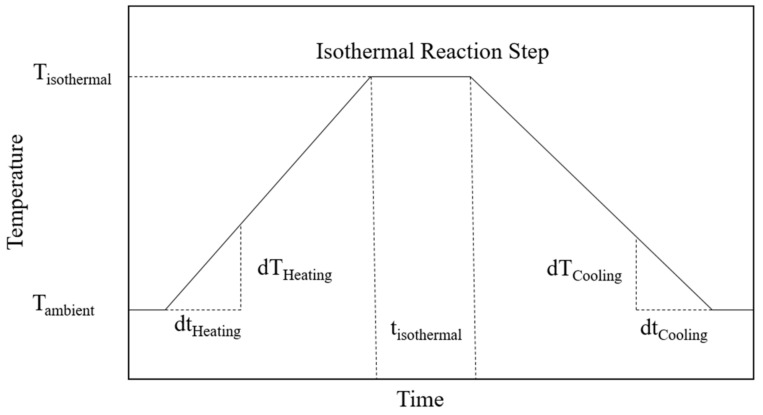
Illustration of the temperature profile and underlying metrices applied for DSC measurements.

Characterization of prepared powder blends is conducted using thermal and rheological characterization. Thermal properties are assessed using differential scanning calorimetry (DSC), applying varying heating rates and isothermal tempering at varying temperatures for characterizing time- and temperature-dependent reaction kinetics of prepared material systems. The underlying temperature profile conducted for thermal analysis by means of DSC is displayed in Figure 3, depicting a constant temperature ramp followed by an isothermal reaction phase and a subsequent cooling phase. Two consecutive heating runs are conducted for quantifying the influence of the previous thermal history on resulting thermal material properties.

DSC measurements conducted for quantifying processing parameters of prepared powder blends are based on a fixed heating and cooling rate of dT/dt = 20 K min^−1^ and a subsequent isothermal phase of t_isothermal_ = 60 s at an isothermal temperature of T_isothermal_ = 240 °C. Further characterizations of temperature-dependent reaction kinetics of a modified material system (ω_pABA_ = 20%), influencing the formation of copolymers and supramolecular structures, are conducted by applying varying isothermal temperatures. An underlying heating rate of dT_Heating_/dt_Heating_ = 200 K min^−1^ is applied for minimizing the influence of the non-isothermal heating process on the thermal history. Subsequent cooling is conducted by applying a cooling rate of dT_Cooling_/dt_Cooling_ = −10 K min^−1^, followed by repeated heating at dT_Heating_/dt_Heating_ = 10 K min^−1^ for characterizing thermal process properties in dependence of the thermal material history. Varied corresponding isothermal temperatures are displayed in Table 2. All differential calorimetric measurements were conducted using a device of type DSC 2500 (TA Instruments, Inc., New Castle, DE, USA).

To allow for quantifying the processing properties of aromatically modified material systems with regard to powder coating and the topology of the resulting powder bed surface, the compressibility of prepared powder blends and the superficial powder bed roughness are assessed. Compressibility measurements, proposed by Hesse et al. (2021) for determining rheological powder properties [35], are conducted using a rheometer of type Discovery HR-20 (TA Instruments, New Castle, DE, USA), applying a maximum axial force of F_Compression_ = 25 N, corresponding to a compression stress of p_Compression_ = 0.08 MPa. Complementary characterizations of the powder bed roughness, depicting a negative correlation with the powder bed density [36], are based on applying optical in situ measurements of coated layers using stripe light projection, applying a recoating speed of *v* = 200 mm s^−1^.

### 3.3. Characterization of Manufactured Components

Mechanical testing is conducted according to DIN EN ISO 527-2:2012, applying a strain rate of 25 mm min^−1^, using an Instron universal testing system (Illinois Tool Works, Glenview, IL, USA) in combination with optical strain measurement. Complementary morphological characterizations include the preparation of polarization micrographs of prepared specimens using a Zeiss AxioImager 2 (Carl Zeiss Microscopy Deutschland GmbH, Jena, Germany) and complementary scanning electron microscopy (SEM) of fractured tensile specimens, applying an SEM of type Zeiss Gemini (Carl Zeiss Microscopy Deutschland GmbH, Jena, Germany). Furthermore, thermal DSC characterizations of manufactured specimens are conducted by applying a constant heating and cooling rate of dT/dt = 10 K min^−1^. Complementary infrared spectroscopic measurements are based on samples extracted from the center of distinct tensile specimens, allowing for determining the presence of molecular structures characteristic of in situ post-condensation reactions. ATR infrared spectroscopy is conducted using an FT-IR device of the type Invenio FT-IR (Bruker Corporation, Billerica, MA, USA) applying a wavelength resolution of 0.5 cm^−1^.

## 4. Results and Discussion

### 4.1. Powder Blend Characteristics

#### 4.1.1. Thermal Powder Blend Properties

**Figure 4:**
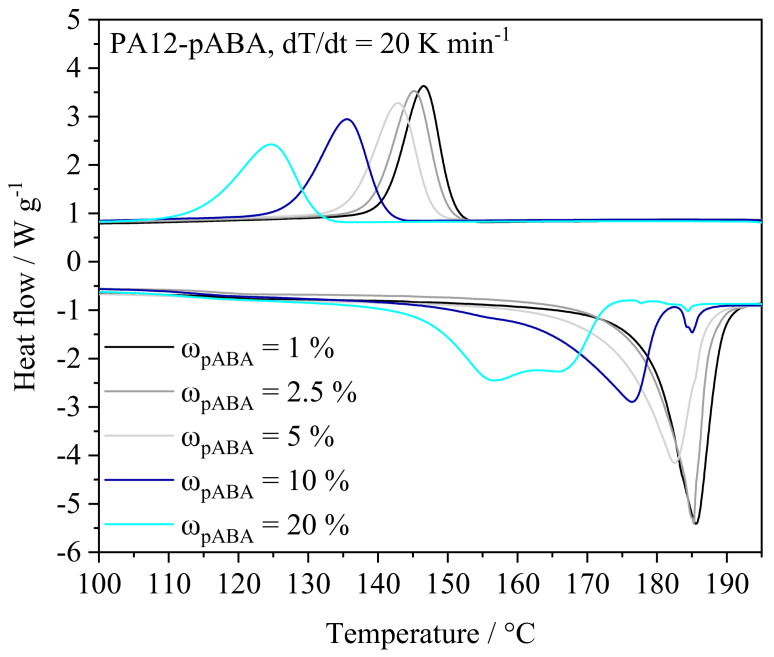
Adapted thermal characteristics of powder blends exhibiting varying mass fractions of pABA, n = 3.

Thermal powder blend properties represent a constituting characteristic in LPBF, determining the applicability and material-specific temperature control with regard to applied isothermal processing conditions. Thermal characteristics, obtained by means of DSC, depict a significant influence of the fraction of p-aminobenzoic acid on the observed melting point, showing a correlation between the fraction of pABA and a corresponding reduction in both melting and crystallization peaks, displayed in Figure 4. Furthermore, increased fractions of pABA lead to a reduced melt and crystallization enthalpy, being correlated with significantly broadened melting and crystallization ranges.

**Figure 5:**
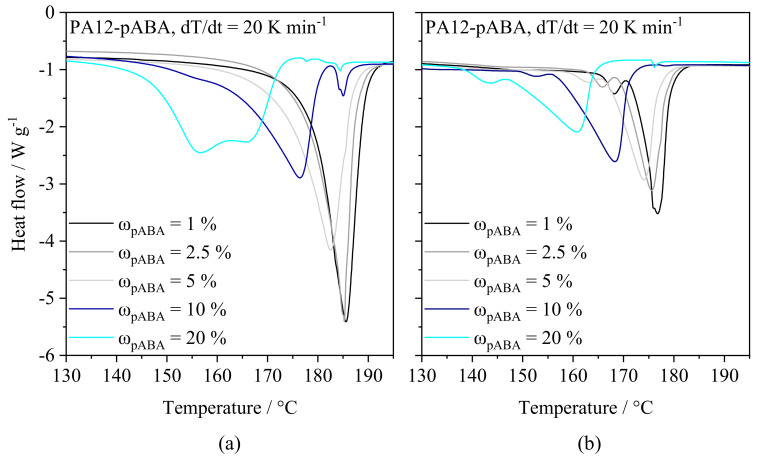
Differential scanning calorimetry of varying fractions of pABA observed in the first (**a**) and second (**b**) heating run, n = 3.

The observed melting point reduction in powder blends is in accordance with observations by Gueche et al. (2021) [37], describing a significant melting point reduction for the application of vinylpyrrolidone–vinyl acetate copolymers modified with aliphatic dicarboxylic acids. Considering time-dependent influences on the resulting thermal material properties, a variation in both the melting process and the crystallization process can be observed. With regard to the second heating run, a decreased melt enthalpy and altered peak temperatures indicate the occurrence of chemical modifications, leading to reduced melting enthalpies. The influence of the thermal history on the melting peak temperature, displayed in Figure 5 can be correlated with experimental findings by Edgar et al. [33], describing reduced melting points of non-isomorphic statistical copolymers.

**Figure 6:**
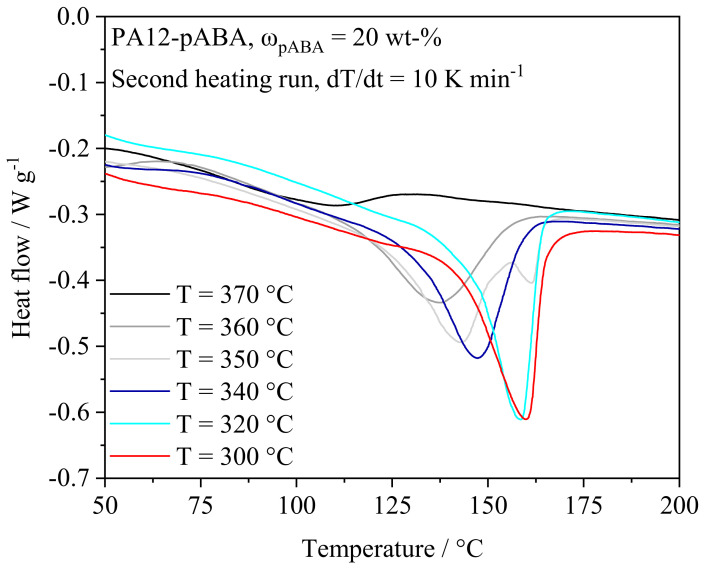
Differential scanning calorimetry of PA12-pABA, ω_pABA_ = 20%, in dependence on the thermal history, t_isothermal_ = 600 s, n = 3.

Considering the molecular structures of 12-aminolauric acid and p-aminobenzoic acid, an indication towards the formation of covalently bound aromatic groups, described for the melt condensation of copolymers [1,2], is evident. Therefore, observed altered thermal properties indicate the occurrence of time- and temperature-dependent auto condensation reactions of PA12-pABA blends. The assumption of the formation of condensed copolymers as well as supramolecular structures [22,23] implicitly influencing crystallization kinetics and observed melting range is further supported based on varying the maximum temperature of the initial heating run. Applying a mass fraction of ω_pABA_ = 20%, a significant influence of the thermal history on thermal material properties can be observed, leading to a diminishing melting enthalpy and reduced melting peak temperatures induced by increased peak temperatures applied within the initial heating run, displayed in Figure 6. Considering an applied isothermal reaction temperature of 370 °C, a vanishing melting peak of the prepared compound at a temperature of 109.9 °C is observed, corresponding to a melting point reduction of 76 K relative to virgin polyamide 12.

Observed temperature-dependent thermal properties depict a correlation with findings described by Zhang et al. (2011) [27], describing the formation of oligo (m-phenyleneisophthalamide) from the corresponding monomers at temperatures ranging from 240 °C to 260 °C. In addition to the formation of non-isomorphic copolymers, the influence of intermolecular hydrogen bonds, implicitly affecting thermal material properties, has been shown to considerably influence thermal properties of cyclodextrins, being associated with combined effects of supramolecular deep eutectic solvents and supramolecular polymerization processes [22]. Therefore, emerging thermal material properties are assumed to rely on the simultaneous occurrence of (auto-)condensation reactions and the formation of supramolecular, hydrogen-bonded structures, both influencing intermolecular interactions.

#### 4.1.2. Rheological Powder Blend Properties

**Figure 7:**
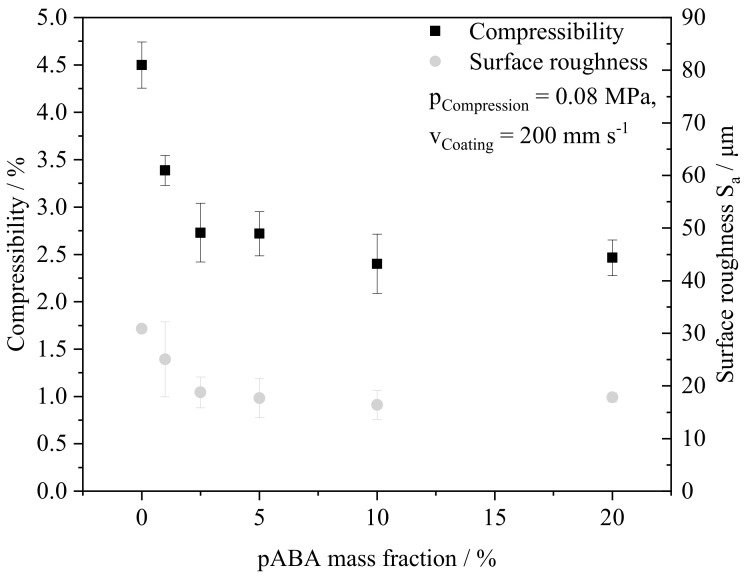
Overview of powder compressibility and powder surface roughness in dependence on contained fractions of pABA, n = 5.

The processability of material systems in the LPBF of polymers considerably depends on the flowability of applied material powders, influencing the powder bed surface roughness [36] and resulting mechanical properties [38] of manufactured components. Based on the determined compressibility of prepared blends, a correlation of the compressibility and the resulting surface roughness can be derived. Increasing fractions of pABA depict a negative correlation with both the compressibility and the optically assessed surface roughness, shown in Figure 7. The observed improvement of rheological processing properties is correlated with an increasing fraction of fumed silica, being added to the fraction of pABA in a constant proportion of 0.5% as a flow agent.

Therefore, increased fractions of pABA allow for improving rheological properties of powder blends, hence facilitating the powder coating process and representing a foundation for the enhanced processability based on an increased powder bed density [36].

### 4.2. Mechanical and Thermal Part Properties

**Figure 8:**
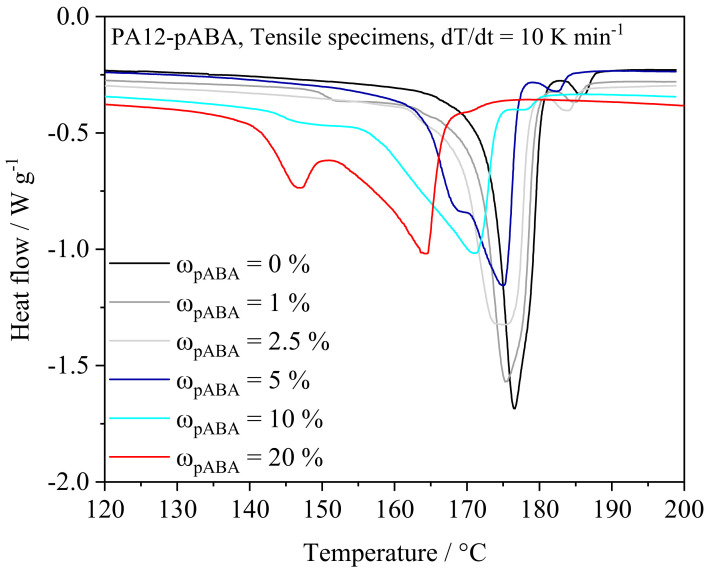
Thermal properties of manufactured tensile specimens assessed using differential scanning calorimetry, n = 3.

Thermal properties assessed by means of DSC exhibit significant deviations of thermal powder blend properties and thermal properties of manufactured tensile specimens. In accordance with the aforementioned DSC measurements of corresponding powder blends, a reduction in the melting enthalpy is interlinked with the thermal history of manufactured specimens. Based on the laser-based additive processing followed by an isothermal holding phase, a significantly reduced melting enthalpy relative to the thermal properties of the underlying powder materials is evident, as displayed in Figure 8. In contrast to thermal part properties observed during repetitive heating runs, a bimodal distribution of emerging thermal properties is evident with regard to a fraction of ω_pABA_ = 20%. Observed melting peaks of T_1_ = 146.6 °C ± 0.26 and T_2_ = 163.8 °C ± 0.53 indicate the formation of a broad range of molecular and supramolecular compositions induced by the laser exposure. Considering the aforementioned DSC measurements, thermal processing conditions in quasi-isothermal LPBF do not initiate the suppression of low-melting crystalline phases. However, in contrast to the ex situ suppression of low-melting crystalline fractions observed in DSC, an occurrence of bimodal melting properties can be observed given a fraction of ω_pABA_ = 20%. Given the aforementioned time- and temperature-dependency of the emergence of low-melting fractions as well as the considerable vanishing of crystalline fractions, detected low-melting fractions are assumed to be interlinked to a limited thermal exposure in LPBF. The negligible occurrence of melting peaks in the region of the melting point of pure pABA indicates a predominantly homogeneous distribution of pABA, obtained by means of powder blending and subsequent laser-based processing.

**Figure 9:**
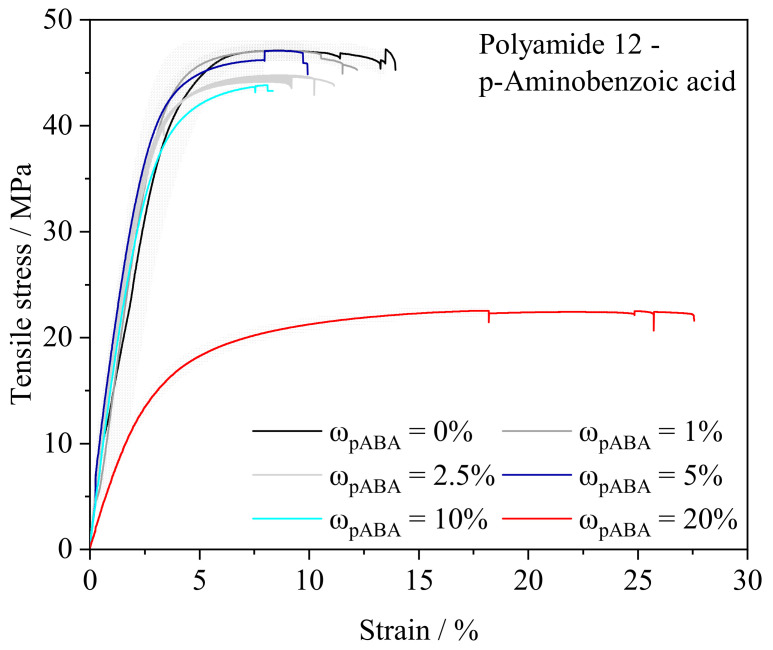
Depiction of the relation of the engineering stress and corresponding engineering strain of manufactured specimens in dependence of the fraction of p-aminobenzoic acid, n = 4, standard deviation indicated as gray shadow.

The mechanical properties of the prepared specimens depict considerable non-linearities, exhibiting slightly reduced mechanical properties of powder blends containing a proportion less than ω_pABA_ = 20% while exhibiting an inconsistent relation of the fraction of pABA and resulting mechanical properties. In contrast, an elevated fraction of ω_pABA_ = 20% leads to a significant reduction in the ultimate tensile strength and the elastic modulus while depicting a considerably enhanced elongation at break of *ε* = 24.65% ± 2.87, representing a significant increase compared with an elongation at break of unmodified polyamide 12 of *ε* = 12.41% ± 1.12, displayed in Figure 9. Furthermore, a fraction of ω_pABA_ = 20% is correlated with a considerably decreased Young’s modulus, being interlinked to the aforementioned improvement of the elongation at break.

Considerably altered mechanical properties are assumed to rely on a reduced crystallinity of manufactured specimens, leading to an enhanced elongation at break while limiting the tensile strength [1]. Therefore, observed mechanical characteristics depict a correlation with aforementioned thermal properties. In this regard, a reduced melt enthalpy of manufactured specimens indicates a reduction in the crystallinity of manufactured samples, being attributed to the observed mechanical characteristics, specifically to a decrease in the elastic modulus and the ultimate tensile stress. Therefore, the observed increase in elongation at break when exceeding a particular fraction of pABA is assumed to be associated with the formation of eutectic supramolecular structures and previously described condensation reactions [11,39], potentially leading to the formation of non-isomorphic polymers.

### 4.3. Part Morphology

**Figure 10:**
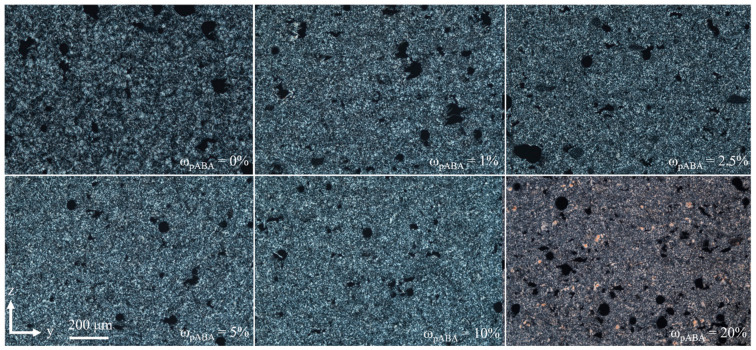
Polarized light micrographs of manufactured tensile specimens in dependence of the fraction of p-aminobenzoic acid.

Regardless of the underlying fraction of p-aminobenzoic acid, formed part morphologies are characterized by the formation of pores, being associated with occasional thermal degradation. The addition of incremental fractions of pABA leads to the formation of reduced spherulite sizes, observed for all applied fractions of pABA, displayed in Figure 10. Exceeding a proportion of 10% of pABA, local morphological variations, observed due to variations in light scattering, are assumed to rely on residual crystals of pABA.

**Figure 11:**
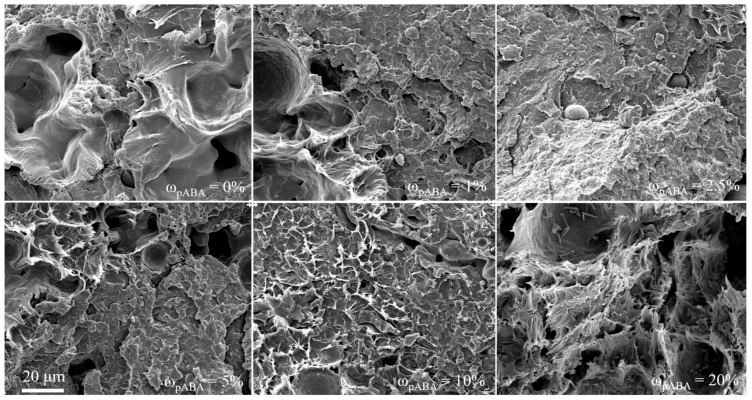
Scanning electron micrographs of fracture surfaces depending on the fraction of pABA.

Complementary scanning electron micrographs, displayed in Figure 11, depict correlations with previously discussed mechanical part properties, showing brittle fracture surfaces at reduced fractions of pABA. In contrast, an elevated elongation at break, evident for a fraction of ω_pABA_ = 20%, is associated with a ductile fracture pattern. Furthermore, the application of elevated fractions of pABA is attributed to the occasional occurrence of recrystallized, undissolved pABA crystals, consistent with polarized light microscopy. Furthermore, the increased formation of pores, observed for a fraction of 20% of pABA, is represented in both polarized light and scanning electron micrographs. Considering previously discussed thermal material properties, the periodic occurrence of pores is assumed to rely on a reduction in the material-specific melt enthalpy. Based on the constant processing parameter, adapted thermal part properties imply a partial thermal degradation due to a material-specific excessive energy input into the melt pool.

### 4.4. Qualitative Characterization of Covalent and Intermolecular Bonds

**Figure 12:**
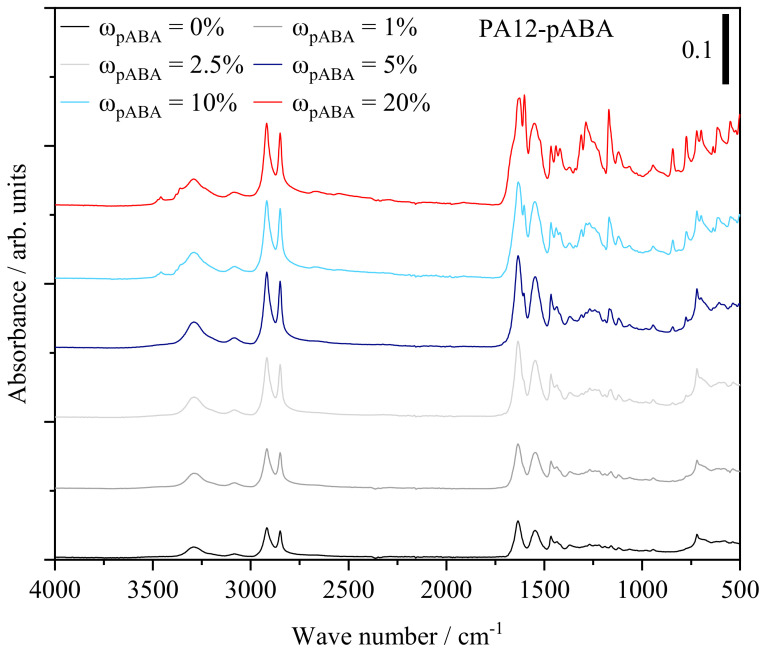
Overview of FTIR spectra of manufactured specimens in dependence on the fraction of pABA; adapted ordinate scaling.

Previously discussed thermal and mechanical part properties indicate the occurrence of molecular variations induced by the partial thermal condensation of p-aminobenzoic acid and polyamide 12 as well as the assumed formation of supramolecular structures, influencing the eutectic material behavior. Based on infrared spectroscopic measurements, considerable variations, depicting a dependency on the fraction of aromatic additive, can be derived, as displayed in Figure 12.

**Figure 13:**
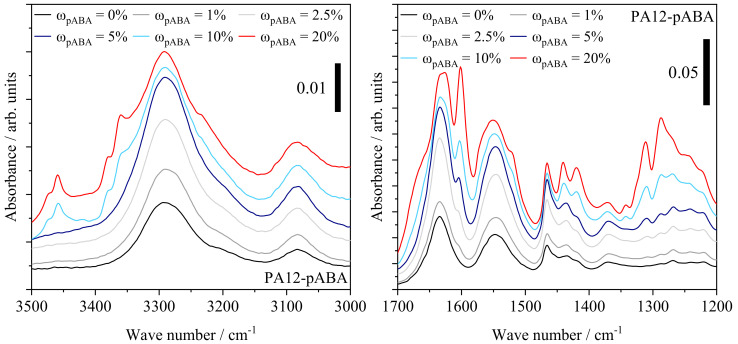
Infrared spectroscopic measurements of manufactured tensile specimens in dependence of the underlying fraction of p-aminobenzoic acid; adapted ordinate scaling.

Increasing fractions of pABA are correlated with an increased representation of hydrogen-bonded N-H stretching of secondary amide bonds observed at 3291 cm^−1^, not observed for the FTIR spectrum of pABA. Similarly, an enhanced absorbance at 1640 cm^−1^, attributed to the amide I band, depicts a dependency on the extent of aromatic additive, governed by vibrational stretching of C=O and C-N bonds, displayed in Figure 13.

**Figure 14:**
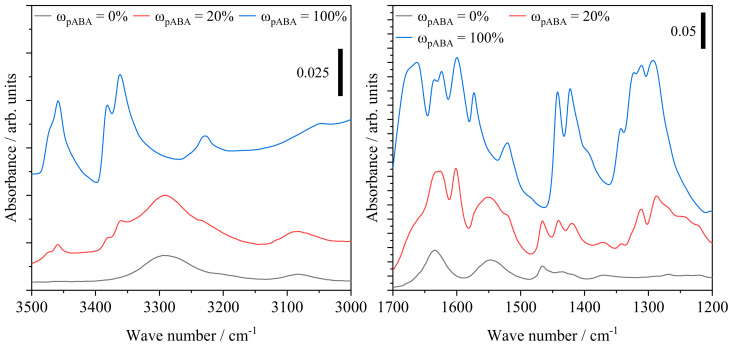
FTIR spectra of exemplary manufactured specimens and the spectrum of pure, unprocessed p-aminobenzoic acid; adapted ordinate scaling.

The amide II band at 1550 cm^−1^, attributed to N-H bending vibration and C-N stretching vibration, depicts a similarly increased representation. In accordance with FTIR investigations of aromatic polyamides, the emergence of a strong signal at 1285 cm^−1^ depicts a correlation with the underlying fraction of aromatic additive, being associated with C-N stretching of aromatic amide bonds similarly represented in the spectrum of the pure additive, evident in Figure 14.

**Figure 15:**
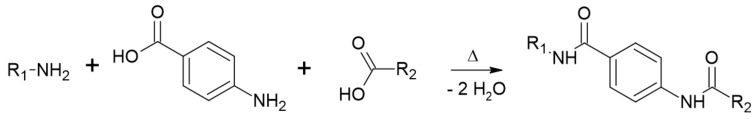
Potential condensation reaction of p-aminobenzoic acid and terminal amino and carboxyl groups of polymeric residues for the formation of secondary amides.

In addition to the increased occurrence of secondary amides, an emerging peak at 3457 cm^−1^ is correlated with N-H stretching of primary amides, indicating the occurrence of non-covalently bound p-aminobenzoic acid at elevated fractions, evident with regard to the spectrum of pure pABA. However, the signal is limited to pABA fractions of 10 wt% and 20 wt%, respectively. Therefore, infrared spectroscopic investigations indicate the occurrence of post-condensation reactions, previously observed in the form of auto-condensation reactions in powder bed fusion of polyamide 12 [11,39,40], hence increasing the proportion of secondary amide groups based on chain-end modifications, displayed in Figure 15.

The spectroscopic determination of primary amides indicates the simultaneous formation of covalent bonds and intermolecular hydrogen bonds in accordance with relevant literature on the formation of non-isomorphic copolymers [41] and supramolecular eutectic polymers [22]. The formation of supramolecular structures can potentially be associated with a characteristic peak at 3460 cm^−1^, interlinked to the intermolecular bonding of OH and C=O-groups [42]. However, given the spectrum of pure pABA, a distinction between peaks originating from intermolecular bonds and amide bands cannot be made, indicating the requirement for further investigations.

For quantifying the proportion of covalent and hydrogen bonds in a filled system containing 20 wt% of pABA, complementary thermogravimetric investigations (dT/dt = 10 K min^−1^) show a mass loss of 13.51 % in a thermal range from 100 °C to 340 °C, indicating a proportion of intermolecularly bound pABA of 67.5%. Therefore, thermal investigations indicate that quasi-isothermal processing conditions lead to a predominant physical interaction of p-aminobenzoic acid and polyamide 12, in accordance with the spectroscopic identification of primary amines. However, the mechanisms and a potential mutual interaction of covalently bound p-aminobenzoic acid and formed supramolecular structures on emerging mechanical and thermal material properties remain subject to further investigations.

## 5. Conclusions

To overcome existing limitations in the range of polymer materials accessible for LPBF, a novel approach for the process-integrated eutectic modification of polyamide 12 in the powder bed fusion of polymers is demonstrated within the present paper. Based on the initial powder blending of polyamide 12 and p-aminobenzoic acid, subsequent laser-based additive processing allows for the in situ modification of the applied polymer, associated with significantly reduced processing temperatures. Thermal investigations and infrared spectroscopy indicate the formation of an adapted chemical structure based on the predominant formation of supramolecular hydrogen-bound structures and the formation of covalently bound secondary amides, promoting the formation of a reduced material crystallinity. Mechanical properties of manufactured tensile bars depict a significant, non-linear dependency on the fraction of p-aminobenzoic acid, exhibiting a reduced tensile strength while displaying a considerably enhanced elongation at break when applying a fraction of 20% aromatic additive.

The modification of thermal, chemical, and mechanical part properties based on binary powder blends represents a promising approach for the targeted adaption of part properties. Given the influence of the thermal history on thermal material properties, the laser-induced local adaption of the chemical structure and the application of other polymers are of considerable interest for future research. Furthermore, the underlying mechanisms regarding the interaction of covalently bound aromatic groups and the formation of supramolecular, hydrogen-bound structures are of great interest.

## Figures and Tables

**Table 1:** Overview of applied mass fractions, corresponding molar fractions of pABA, and applied material-specific isothermal build chamber temperatures.

pABA Mass Fraction/%	pABA Molar Fraction/mol.-%	Isothermal Build Chamber Temperature/°C
0.0	0.00	172.0
1.0	1.56	170.0
2.5	3.87	167.5
5.0	7.63	155.0
10	14.86	144.5
20	28.19	141.5

**Table 2:** Isothermal temperature values and corresponding isothermal holding times applied for characterizing high-temperature reaction kinetics of aromatically modified Polyamide 12.

Isothermal Temperature/°C	Isothermal Reaction Time/s
300	600
320	600
340	600
350	600
360	600
370	600

## Data Availability

The data presented in this study are not publicly available due to ongoing research in this field.

## References

[1] Rwei S.-P., Ranganathan P., Chiang W.-Y., Lee Y.-H. (2018). Synthesis of Low Melting Temperature Aliphatic-Aromatic Copolyamides Derived from Novel Bio-Based Semi Aromatic Monomer. Polymers.

[2] Wolffs M., Cotton L., Kolkman A.J., Rulkens R. (2021). New sustainable alternating semi-aromatic polyamides prepared in bulk by direct solid-state polymerization. Polym. Int..

[3] Peng S., Peng L., Yi C., Zhang W., Wang X. (2018). A novel synthetic strategy for preparing semi-aromatic components modified polyamide 6 polymer. J. Polym. Sci. Part A Polym. Chem..

[4] Higashihara T., Zhang C., Tsukuda A., Ochi T., Ueda M. (2012). Direct synthesis and melt-drawing property of aramids by bulk polycondensation of isophthalic acid with m-phenylenediamine and 3,4′-oxydianiline. J. Appl. Polym. Sci..

[5] Yamazaki N., Matsumoto M., Higashi F. (1975). Studies on reactions of the N-phosphonium salts of pyridines. XIV. Wholly aromatic polyamides by the direct polycondensation reaction by using phosphites in the presence of metal salts. J. Polym. Sci. Polym. Chem. Ed..

[6] Shibasaki Y., Abe Y., Sato N., Fujimori A., Oishi Y. (2010). Direct condensation polymerization of N-alkylated p-aminobenzoic acid and packing of rigid-rod main chains with flexible side chains. Polym. J..

[7] Greiner S., Jaksch A., Cholewa S., Drummer D. (2021). Development of material-adapted processing strategies for laser sintering of polyamide 12. Adv. Ind. Eng. Polym. Res..

[8] Drummer D., Greiner S., Zhao M., Wudy K. (2019). A novel approach for understanding laser sintering of polymers. Addit. Manuf..

[9] Soldner D., Greiner S., Burkhardt C., Drummer D., Steinmann P., Mergheim J. (2020). Numerical and experimental investigation of the isothermal assumption in selective laser sintering of PA12. Addit. Manuf..

[10] Soldner D., Steinmann P., Mergheim J. (2021). Modeling crystallization kinetics for selective laser sintering of polyamide 12. GAMM-Mitteilungen.

[11] Wudy K., Drummer D. (2019). Aging effects of polyamide 12 in selective laser sintering: Molecular weight distribution and thermal properties. Addit. Manuf..

[12] El-Mazry C., Ben Hassine M., Correc O., Colin X. (2013). Thermal oxidation kinetics of additive free polyamide 6-6. Polym. Degrad. Stab..

[13] Pliquet M., Rapeaux M., Delange F., Bussiere P.O., Therias S., Gardette J.L. (2021). Multiscale analysis of the thermal degradation of polyamide 6,6: Correlating chemical structure to mechanical properties. Polym. Degrad. Stab..

[14] Lanzl L., Wudy K., Greiner S., Drummer D. (2019). Selective laser sintering of copper filled polyamide 12: Characterization of powder properties and process behavior. Polym. Compos..

[15] Lanzl L., Wudy K., Drummer D. (2020). The effect of short glass fibers on the process behavior of polyamide 12 during selective laser beam melting. Polym. Test..

[16] Roda A., Matias A.A., Paiva A., Duarte A.R.C. (2019). Polymer Science and Engineering Using Deep Eutectic Solvents. Polymers.

[17] Janicka P., Kaykhaii M., Płotka-Wasylka J., Gębicki J. (2022). Supramolecular deep eutectic solvents and their applications. Green Chem..

[18] Hansen B.B., Spittle S., Chen B., Poe D., Zhang Y., Klein J.M., Horton A., Adhikari L., Zelovich T., Doherty B.W. (2021). Deep Eutectic Solvents: A Review of Fundamentals and Applications. Chem. Rev..

[19] Stefanovic R., Ludwig M., Webber G.B., Atkin R., Page A.J. (2017). Nanostructure, hydrogen bonding and rheology in choline chloride deep eutectic solvents as a function of the hydrogen bond donor. Phys. Chem. Chem. Phys..

[20] Ibrahim R.K., Hayyan M., AlSaadi M.A., Ibrahim S., Hayyan A., Hashim M.A. (2019). Physical properties of ethylene glycol-based deep eutectic solvents. J. Mol. Liq..

[21] Abbott A.P., Capper G., Davies D.L., Rasheed R.K., Tambyrajah V. (2003). Novel solvent properties of choline chloride/urea mixtures. Chem. Commun..

[22] Wu S., Cai C., Li F., Tan Z., Dong S. (2020). Deep Eutectic Supramolecular Polymers: Bulk Supramolecular Materials. Angew. Chem. Int. Ed..

[23] El Achkar T., Moufawad T., Ruellan S., Landy D., Greige-Gerges H., Fourmentin S. (2020). Cyclodextrins: From solute to solvent. Chem. Commun..

[24] De Lacalle J.L., Gallastegui A., Olmedo-Martínez J.L., Moya M., Lopez-Larrea N., Picchio M.L., Mecerreyes D. (2023). Multifunctional Ionic Polymers from Deep Eutectic Monomers Based on Polyphenols. ACS Macro Lett..

[25] Wittmann J.C., Manley R.S.J. (1977). Polymer–monomer binary mixtures. I. Eutectic and epitaxial crystallization in poly (ε-caprolactone)–trioxane mixtures. J. Polym. Sci. Polym. Phys. Ed..

[26] Papaspyrides C.D., Porfyris A.D., Vouyiouka S., Rulkens R., Grolman E., Poel G.V. (2016). Solid state polymerization in a micro-reactor: The case of poly (tetramethylene terephthalamide). J. Appl. Polym. Sci..

[27] Zhang C., Shoji Y., Higashihara T., Tsukuda A., Ochi T., Ueda M. (2011). Synthesis of poly (m-phenyleneisophthalamide) by solid-state polycondensation of isophthalic acid with m-phenylenediamine. J. Polym. Sci. Part A Polym. Chem..

[28] Jeyakumar A., Goossens H., Noordover B., Prusty M., Scheibitz M., Koning C. (2013). Polyamide-6,6-based blocky copolyamides obtained by solid-state modification. J. Polym. Sci. Part A Polym. Chem..

[29] Papaspyrides C.D., Vouyiouka S.N., Bletsos I.V. (2006). New aspects on the mechanism of the solid state polyamidation of PA 6,6 salt. Polymer (Guildf)..

[30] Papaspyrides C.D., Porfyris A.D., Rulkens R., Grolman E., Kolkman A.J. (2016). The effect of diamine length on the direct solid state polycondensation of semi-aromatic nylon salts. J. Polym. Sci. Part A Polym. Chem..

[31] Porfyris A., Vouyiouka S., Papaspyrides C., Rulkens R., Grolman E., Vanden Poel G. (2016). Investigating alternative routes for semi-aromatic polyamide salt preparation: The case of tetramethylenediammonium terephthalate (4T salt). J. Appl. Polym. Sci..

[32] Zhang C. (2018). Progress in semicrystalline heat-resistant polyamides. e-Polymers.

[33] Edgar O.B., Hill R. (1952). The p-phenylene linkage in linear high polymers: Some structure–property relationships. J. Polym. Sci..

[34] Endo T., Higashihara T. (2022). Direct Synthesis of Thermally Stable Semiaromatic Polyamides by Bulk Polymerization Using Aromatic Diamines and Aliphatic Dicarboxylic Acids. ACS Omega.

[35] Hesse N., Winzer B., Peukert W., Schmidt J. (2021). Towards a generally applicable methodology for the characterization of particle properties relevant to processing in powder bed fusion of polymers–From single particle to bulk solid behavior. Addit. Manuf..

[36] Sillani F., Schiegg R., Schmid M., MacDonald E., Wegener K. (2022). Powder Surface Roughness as Proxy for Bed Density in Powder Bed Fusion of Polymers. Polymers.

[37] Gueche Y.A., Sanchez-Ballester N.M., Bataille B., Aubert A., Leclercq L., Rossi J.-C., Soulairol I. (2021). Selective Laser Sintering of Solid Oral Dosage Forms with Copovidone and Paracetamol Using a CO (2) Laser. Pharmaceutics.

[38] Beitz S., Uerlich R., Bokelmann T., Diener A., Vietor T., Kwade A. (2019). Influence of Powder Deposition on Powder Bed and Specimen Properties. Materials.

[39] Benz J., Bonten C. (2019). Temperature induced ageing of PA12 powder during selective laser sintering process. AIP Conf. Proc..

[40] Drummer D., Wudy K., Drexler M. (2015). Modelling of the aging behavior of polyamide 12 powder during laser melting process. AIP Conf. Proc..

[41] Jeyakumar A. (2012). Solid-State Modification of Polyamide-6,6. Ph.D. Thesis.

[42] Guo L., Sato H., Hashimoto T., Ozaki Y. (2010). FTIR Study on Hydrogen-Bonding Interactions in Biodegradable Polymer Blends of Poly (3-hydroxybutyrate) and Poly (4-vinylphenol). Macromolecules.

